# Evaluation of functional outcome of bilateral kidney tumors after sequential surgery

**DOI:** 10.1186/s12885-021-08324-3

**Published:** 2021-05-24

**Authors:** Jung Kwon Kim, Hwanik Kim, Hakmin Lee, Jong Jin Oh, Sangchul Lee, Sung Kyu Hong, Cheol Kwak, Seok-Soo Byun

**Affiliations:** 1 Department of Urology, Seoul National University Bundang Hospital (SNUBH), 166 Gumi-Ro, Bundang-gu, Seongnam-si, Gyeonggi-do 463-707 Korea; 2 Department of Urology, Seoul National University College of Medicine, 103 Daehak-ro, Jongno-gu, Seoul, 03080 Korea; 3 Department of Urology, Seoul National University Hospital (SNUH), Seoul, Korea

**Keywords:** Bilaterality, Functional outcomes, Renal cell carcinoma, Partial nephrectomy, Sequence of surgery

## Abstract

**Background:**

There are limited data concerning patients treated with sequential bilateral kidney surgery. Current guidelines still lack an optimal surgical sequencing approach. We evaluated renal functional outcomes after sequential partial nephrectomy (PN) and radical nephrectomy (RN) in patients with bilateral renal cell carcinoma (RCC).

**Methods:**

A propensity score matched cohort of 267 patients (synchronous bilateral RCCs, *N* = 44 [88 lesions]; metachronous bilateral, *N* = 45 [90 lesions]; unilateral, *N* = 178) from two tertiary institutions were retrospectively analyzed. Synchronous bilateral RCCs were defined as diagnosis concomitantly or within 3 months of former tumor. Renal functional outcomes were defined as estimated glomerular filtration rate (eGFR) changes and de novo chronic kidney disease (CKD, stage ≥3) after surgery. Renal functional outcomes and clinical factors predicting de novo CKD were assessed using descriptive statistics and Cox regression analysis.

**Results:**

In subgroup of bilateral RCCs, patients underwent sequential PN (*N* = 48), PN followed by RN (*N* = 8), or RN followed by PN (*N* = 25). Final postoperative estimated glomerular filtration rates (eGFRs) were 79.4, 41.4, and 61.2 ml/minute/1.73 m2, respectively (*p* = 0.003). There were significant differences in eGFR decline from baseline and de novo chronic kidney disease (CKD stage ≥ III) among groups, with PN followed by RN group showing the worst functional outcomes (all *p* <  0.05). Moreover, sequential PN subgroup in bilateral RCC showed significantly higher rate of de novo CKD than unilateral RCC group (13.8% vs. 6.9%, *p* = 0.016). On multivariate analysis, hypertension (*p* = 0.010) and surgery sequence (PN followed by RN, *p* <  0.001) were significant predictors of de novo CKD.

**Conclusions:**

The surgery sequence should be prudently determined in bilateral renal tumors. PN followed by RN showed a negative impact on renal functional preservation. Nephron-sparing surgery should be considered for all amenable bilateral RCCs.

**Supplementary Information:**

The online version contains supplementary material available at 10.1186/s12885-021-08324-3.

## Introduction

It has been estimated that 73,800 new cases of cancers of the kidney and renal pelvis will be diagnosed in the United States in 2020 and that 14,800 people will die of them [1]. Approximately 85% of all kidney tumors are renal cell carcinoma (RCC). RCC occurs in bilateral kidneys, including synchronous RCC (diagnosed concomitantly or within 3 months of the former tumor) and metachronous RCC (tumor diagnosed 3 months after former tumor detection) in approximately 5% of all RCC patients [2–5]. Due to the relative rarity of bilateral presentation, even until now, there are limited data in the literature concerning patients treated with sequential bilateral kidney surgery. Few literatures have evaluated the functional impact of bilateral kidney surgery and how functional outcomes are influenced by tumor characteristics, modality selection, and patient-related risk factors [6–9].

Nevertheless, several studies have elucidated renal functional outcomes in patients with bilateral synchronous tumors who have undergone sequential bilateral kidney surgery. Simmons et al. have demonstrated that bilateral partial nephrectomy (PN) is associated with significantly improved estimated glomerular filtration rate (eGFR) compared to radical nephrectomy (RN) [6]. Packiam et al. have reported that patients for nonmetastatic bilateral synchronous tumors who have received simultaneous PN show lower mean postoperative 3 months (− 6% vs. -24%, *p* = 0.015) and median postoperative 12 months (− 4% vs. -22%, *p* <  0.001) reduction in eGFR compared to staged (within 6 months) PN, respectively [7]. Singer et al. have observed that nephron-sparing surgery (NSS) enables dialysis to be avoided in more than 95% of patients [8]. Another study reported average 28.9% eGFR decline after treatment of both kidneys with 608-day follow-up (59 → 41.9 ml/min/1.73 m^2^) [9].

However, few studies have compared long term functional outcomes according to procedure sequence. Current guidelines still lack an optimal surgical sequencing approach [10–12]. Therefore, the objective of this study was to evaluate renal functional outcomes after sequential PN and RN in patients with bilateral RCC.

## Materials and methods

### Ethics statement

The Institutional Review Board of the Seoul National University Bundang Hospital approved this study (Approval number: B-2007-625-102). As the present study was carried out retrospectively, written informed consent from patients was waived. Personal identifiers were completely removed and data were analyzed anonymously. Our study was conducted according to ethical standards recommended by the 1964 Declaration of Helsinki and its later amendments.

### Study cohort

**Fig. 1:**
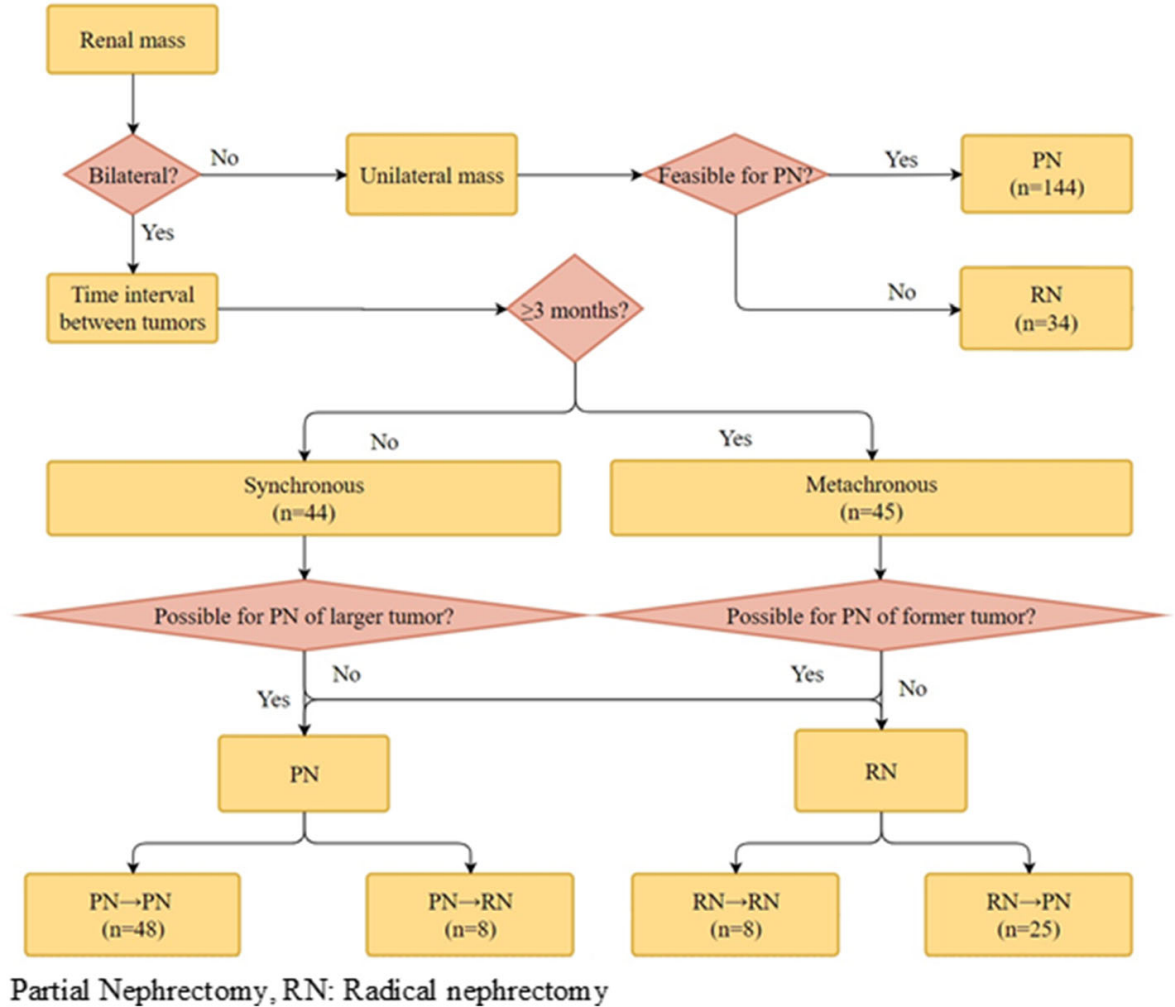
Treatment algorithm

From June 2003 to March 2018, a total of 267 patients (synchronous bilateral RCCs, *N* = 44 [88 tumor lesions]; metachronous bilateral RCCs, *N* = 45 [90 lesions]; unilateral RCCs, *N* = 178) from two tertiary institutions (SNUH and SNUBH) were included in this study. Synchronous bilateral RCCs were defined as diagnoses concomitantly or within 3 months of the former tumor. Metachronous bilateral RCCs were defined as tumor diagnoses at intervals of at least 3 months. All included patients underwent surgery by open, laparoscopic, or robotic approach which was left to surgeon discretion, with curative intent in two centers [4–7]. Main operating surgeons determine the sequence of surgery under consideration of patients’ clinical setting and multidisciplinary consultation. In general, treatment for the larger tumor was implemented first to allow better tumor control and the contralateral kidney to assist in recovery of renal function (Fig. 1). Variation in time between surgeries can be attributed to differences in recovery speed from initial surgery, patients’ willingness to receive subsequent surgery, and preoperative general medical conditions. Patients were excluded if they had non-RCC histology or hereditary kidney diseases (e.g., the von Hippel-Lindau [VHL] disease, autosomal dominant polycystic kidney disease [ADPKD], Birt-Hogg-Dubé [BHD] syndrome, and so on). Clinical data in medical records were retrospectively reviewed [13].

### Acquisition and definition of data

Clinical perioperative variables included age, gender, body mass index (BMI), past medical history (including diabetes mellitus [DM], hypertension [HTN], and chronic kidney disease [CKD]), symptoms at presentation, Eastern Cooperative Oncology Group (ECOG) performance status, and types of surgery (PN vs. RN). Pathological parameters including histological type according to the World Health Organization (WHO) classification system, pathologic stage according to the 8th edition of American Joint Committee guidelines, Fuhrman nuclear grade, and positive surgical margin were also evaluated [14, 15]. Tumor sizes are defined as the longest length of axes measured from pathologic specimen after surgery. Ischemic time is defined as warm ischemia time from first arterial clamping to first arterial unclamping. These are all monitored at the time of operation by main operating surgeon. Renal functional outcomes were defined as estimated glomerular filtration rate (eGFR) changes and incidence of de novo CKD (stage ≥3) after surgery. eGFR changes at specific postoperative period was evaluated as follows: differences between postoperative 1 week eGFR and preoperative baseline eGFR, between postoperative 1 month eGFR and baseline eGFR, between postoperative 1 year eGFR and baseline eGFR, and between latest eGFR and baseline eGFR. Renal functional evaluation was conducted using serum creatinine values obtained immediately before initial surgery, immediately before second surgery, and 3 to 6 months after the second surgery. Modification of Diet in Renal Disease equation was used to calculate eGFR [16]. CKD staging was conducted according to the National Kidney Foundation Disease Outcomes Quality Initiative Clinical Practice Guidelines [17].

### Follow-up protocol

According to institutional standardized postoperative protocol, patients were generally followed-up after surgery at least every 6 months in the first year, annually during the next 4 years, and every 2 years thereafter. Follow-up protocols consisted of blood test including serum creatinine, computed tomography or magnetic resonance imaging studies, and chest radiography.

### Statistical analyses

Clinicopathological characteristics were compared between unilateral and bilateral RCC groups using Chi-squared test or Kruskal-Wallis test for categorical variables and one-way analysis of variance, independent t-test or Mann-Whitney U test for continuous variables as appropriate. Univariate and multivariate Cox-proportional hazard regression analyses were performed to evaluate significant variables associated with de novo CKD within postoperative 1 year and along entire follow-up period. Proportional hazard assumptions were evaluated using Schoenfeld test and log-log plot. The univariate results were used to determine the candidate variables for the final multivariate model in a backward model selection process. In all variables remaining in the final multivariate analysis, the *p* value was set to 0.05. Age, hypertension, pathologic tumor size, surgery sequence, and tumor chronology (only for Table 6) are variables included in the multivariate analysis at both Tables 5 and 6. In addition, eGFR changes and clinical factors predicting de novo CKD in the propensity score matched cohort were assessed using descriptive statistics and Cox regression analysis [18]. The propensity score matching (PSM) was used to control other confounding factors when analyzing renal functional outcomes of unilateral and bilateral RCCs. The propensity scores matched using the nearest neighbor, 1:1 pair matching within 0.2 standard deviations (SDs) of the logit of the propensity score. By calculating the standardized differences of the means or proportions of each covariate after matching, baseline covariates with standardized differences < 0.1 indicated a good balance between groups. Age, sex, DM, HTN, CKD, BMI, ECOG performance status, baseline eGFR, surgical type, expected blood loss, ischemic time, and pathologic tumor size was used as covariates in the PSM process. Subgroup analysis of bilateral RCC among synchronous and metachronous RCCs was also performed. All statistical analyses were performed using commercially available software IBM SPSS Statistics ver. 21.0 (Armonk, NY, USA and the statistical package for R, ver. 2.13.2 (R Foundation for Statistical Computing [http://www.r-project.org/]). Two-sided *p* values < 0.05 were considered statistically significant.

## Results

Baseline characteristics between bilateral RCC group and unilateral RCC group are detailed in Table 1. Pre-propensity table for the cohort before matching are detailed in supplementary Table 1. There were no significant differences in baseline characteristics between groups after propensity score matching. Median time between surgeries in bilateral RCC group was 146.0 days (37.5 days for synchronous and 1899.0 days for metachronous subgroup). In the bilateral RCC cohort, subgroup comparative analysis for renal functional outcome (PN followed by PN vs. PN followed by RN vs. RN followed by PN group) was conducted (Table 2). Patients underwent sequential PN (*n* = 48), PN followed by RN (*n* = 8), or RN followed by PN (*n* = 25). Patients with sequential RN were excluded in the subgroup analysis due to intuitively prominent renal function deterioration compared to other groups. The mean eGFR follow-up duration was 65.6 ± 47.6 months (range, 0–166 months). Final eGFR after bilateral surgery was 79.4 ± 33.9, 41.4 ± 28.3, and 61.2 ± 29.8 ml/minute/1.73 m^2^ in these three groups, respectively (*p* = 0.003). There were significant differences in eGFR decline from baseline and de novo CKD among groups, with PN followed by RN group showing the worst functional outcomes (all *p* <  0.05). PN followed by PN group had significantly higher latest eGFR (*p* = 0.003), less eGFR decline from baseline during the entire postoperative follow-up period (all *p* <  0.05). They had also significantly less de novo CKD stage ≥3 occurred within a year after 2nd operation (*p* = 0.036), less de novo CKD stage ≥3 occurred after the one-year minimal follow-up period after 2nd operation (median/mean follow-up period: 54.0/65.6 months, *p* <  0.001), and less total number of patients with CKD at the last follow-up than other groups. RN followed by PN group had significantly less eGFR decline than PN followed by RN group at postoperative 1 week (*p* = 0.031) and the latest follow-up period (*p* = 0.001) from baseline.

**Table 1:** Baseline characteristics after propensity score matching

Variables	Bilateral RCC (*N* = 178 lesions of 89 patients)		Unilateral RCC (*N* = 178 lesions of 178 patients)	*P* value^§^
	Synchronous lesion (*N* = 88)	Metachronous lesion (*N* = 90)		
Age, mean (SD)	54.0 ± 12.7	55.9 ± 12.9	54.2 ± 13.4	0.549
Sex, male, N (%)	74 (84.1%)	74 (82.2%)	142 (79.8%)	0.856
DM, yes, N (%)	17 (19.3%)	18 (20.0%)	22 (16.7%)	0.792
HTN, yes, N (%)	47 (53.4%)	42 (46.7%)	89 (50.0%)	0.564
BMI, mean (SD)	25.0 ± 3.7	24.2 ± 3.1	24.7 ± 3.0	0.367
ECOG performance status, N (%)				0.415
≤ 1	86 (97.7%)	86 (95.6%)	167 (93.8%)	
≥ 2	2 (2.3%)	4 (4.4%)	11 (6.2)	
Surgical type, N (%) (Open / Laparoscopic / Robotic)	37 (42.0%) / 13 (14.8%) / 38 (43.2%)	44 (48.9%) / 9 (10.0%) / 37 (41.1%)	94 (52.8%) / 11 (6.2%) / 73 (41.0%)	0.183
EBL, ml, mean (SD)	249.1 ± 107.5	259.0 ± 217.9	226.1 ± 261.5	0.878
Ischemic time, min, mean (SD)^a^	21.5 ± 9.8	22.2 ± 10.7	21.4 ± 16.3	0.904
Pathologic tumor size, mm, mean (SD)	33.5 ± 34.8	32.1 ± 27.6	32.8 ± 23.3	0.949
Baseline eGFR, ml/min/1.73 m^2^, mean (SD)	71.6 ± 32.5	76.0 ± 25.4	78.6 ± 22.3	0.224
Baseline CKD, stage ≥3	28 (31.8%)	16 (17.8%)	39 (21.9%)	0.068

^§^ Evaluates differences among all 3 groups

^a^ Partial nephrectomy only

**Table 2:** Functional outcome of bilateral RCC cohort regardless of time interval between tumors

Variables	PN ➔ PN (*N* = 48)	PN ➔ RN (*N* = 8)	RN ➔ PN (*N* = 25)	*P* value ^§^
Preoperative eGFR, ml/min/1.73 m^2^, mean (SD) (before 1st surgery)	84.7 ± 25.4	83.1 ± 19.6	79.6 ± 33.4	0.759
Latest eGFR, ml/min/1.73 m^2^, mean (SD)	79.4 ± 33.9	41.4 ± 28.3	61.2 ± 29.8	0.003
		*p* = 0.108 ^a^		
eGFR decline from baseline, mean (SD)				
Postoperative 1 week (after 2nd surgery)	6.9 ± 18.5	28.5 ± 18.2	10.6 ± 16.5	0.009
		*p* = 0.031 ^a^		
Postoperative 1 month (after 2nd surgery)	5.1 ± 15.0	28.7 ± 23.2	9.9 ± 13.2	0.001
		*p* = 0.058 ^a^		
Postoperative 1 year (after 2nd surgery)	5.8 ± 17.5	29.4 ± 24.2	31.3 ± 6.3	0.018
		*p* = 0.035 ^a^		
Latest	−0.64 ± 22.5	30.5 ± 26.6	−2.7 ± 21.3	0.001
		*p* = 0.001 ^a^		
De novo CKD, stage≥3				
Baseline CKD, before 1st surgery	8 (16.7%)	1 (12.5%)	5 (20.0%)	0.874
Baseline CKD, before 2nd surgery	6 (12.5%)	1 (12.5%)	9 (36.0%)	0.049
De novo, ≤ 1 year after 2nd surgery	9 (18.8%)	4 (50%)	5 (20%)	0.036
De novo, > 1 year after 2nd surgery	0 (0%)	2 (25.0%)	1 (4.0%)	< 0.001
Total number of CKD patients last follow-up	15 (31.3%)	7 (87.5%)	15 (60%)	< 0.001

*SD* Standard Deviation

eGFR follow-up duration (mean ± SD): 65.6 ± 47.6 months (range, 0–166 months)

8 patients with RN followed by RN were excluded from the analysis

^§^ Evaluates differences among all surgery groups at specific points

^a^ Evaluates differences between ‘PN → RN’ group and ‘RN → PN’ group

We also performed another subgroup analysis between bilateral synchronous RCC (PN followed by PN) subgroup and unilateral RCC (single PN) subgroup (Table 3). The bilateral synchronous RCC (PN followed by PN) subgroup had no significant difference in eGFR decline at the latest follow-up period from baseline compared to the unilateral RCC (single PN) subgroup (*p* = 0.770), although it had higher de novo CKD rate until postoperative 1 year and during the entire follow-up period (13.8% vs. 6.9%, *p* = 0.016). Additional subgroup analysis between bilateral synchronous and metachronous RCC (PN followed by PN) subgroups revealed no significant differences in variables among groups (Table 4).

**Table 3:** Comparative analysis between bilateral synchronous tumor subgroup (PN followed by PN) and unilateral RCC (single PN)

Variables	Bilateral (*N* = 58 lesions of 29 patients)	Unilateral (*N* = 144 lesions of 144 patients)	*P* value
Preoperative eGFR, ml/min/1.73 m^2^, mean (SD)	78.2 ± 24.3	82.2 ± 22.9	0.106
^a^Latest eGFR, ml/min/1.73 m^2^, mean (SD)	80.2 ± 22.7	83.1 ± 20.4	0.182
eGFR decline from baseline, mean (SD)			
Postoperative 1 week	7.2 ± 16.1	−3.5 ± 15.4	< 0.001
Postoperative 1 month	3.5 ± 14.6	−1.7 ± 15.5	0.037
Postoperative 1 year	5.3 ± 17.3	0.9 ± 16.3	0.099
^b^Latest	−2.0 ± 23.8	−1.0 ± 18.5	0.770
De novo CKD, ≤ 1 year	8 (13.8%)	8 (7.5%)	0.022
De novo CKD, total	8 (13.8%)	10 (6.9%)	0.016

^a^Latest eGFR: The very last estimated Glomerular Filtration Rate measured during the follow-up period

^b^Latest: The last time a patient was followed-up and evaluated eGFR

**Table 4:** Comparative analysis between PN followed by PN subgroups (synchronous vs. metachronous) [latter surgery]

Variables	Synchronous (*N* = 29 patients)	Metachronous (*N* = 19 patients)	*P* value
Preoperative eGFR, ml/min/1.73 m^2^, mean (SD)	76.5 ± 22.4	82.1 ± 25.1	0.434
^a^Latest eGFR, ml/min/1.73 m^2^, mean (SD)	80.2 ± 24.0	78.1 ± 31.2	0.247
eGFR decline from baseline, mean (SD)			
Postoperative 1 week	6.5 ± 18.0	7.4 ± 19.7	0.871
Postoperative 1 month	3.0 ± 14.9	8.4 ± 15.1	0.230
Postoperative 1 year	3.5 ± 17.5	9.3 ± 17.5	0.266
^b^Latest	−3.7 ± 24.1	4.0 ± 19.5	0.250
De novo CKD, stage ≥3			
Baseline CKD, Before 1st surgery	7 (24.1%)	1 (5.3%)	0.123
Baseline CKD, Before 2nd surgery	5 (17.2%)	1 (5.3%)	0.381
De novo, ≤ 1 year after 2nd surgery	5 (17.2%)	4 (21.1%)	1.000
De novo, > 1 year after 2nd surgery	0 (0%)	0 (0%)	1.000
Total number of pts. with CKD at last follow-up	10 (34.5%)	5 (26.3%)	0.751

^a^Latest eGFR: The very last estimated Glomerular Filtration Rate measured during the follow-up period

^b^Latest: The last time a patient was followed-up and evaluated eGFR

Multivariate analysis for the prediction of de novo CKD until postoperative 1 year revealed that hypertension (Hazard ratio [HR]: 2.159, 95% Confidence Interval [CI]: 1.233–3.783, *p* = 0.007), pathologic tumor size at 1st surgery (HR: 1.012, 95% CI: 1.006–1.024, *p* = 0.010) and PN followed by RN sequence (HR: 1.837, 95% CI: 1.028–3.635, *p* = 0.007) were significant factors (Table 5). A multivariate analysis for the prediction of de novo CKD during the entire period revealed that hypertension (HR: 1.905, 95% CI: 1.172–3.265, *p* = 0.010), PN followed by RN sequence (HR: 1.888, 95% CI: 1.088–4.055, *p* <  0.001), and metachronous RCC (HR: 2.682, 95% CI: 1.032–6.973, *p* = 0.043) were significant predictive factors (Table 6). The difference in time interval between tumor occurrences in metachronous RCC was not significantly related to de novo CKD incidence (*p* = 0.083 for de novo CKD within postoperative 1 year and *p* = 0.056 for de novo CKD during the entire follow-up period).

**Table 5:** Multivariate Cox regression analyses of variables associated with de novo CKD within postoperative one year

	Univariate analysis			Multivariate analysis		
	HR	95% CI	*P* value	HR	95% CI	*P* value
Age (years)	1.032	1.009–1.056	0.001	1.036	1.011–1.062	0.005
BMI	1.011	0.924–1.106	0.810			
Sex, male	0.711	0.348–1.454	0.350			
DM	1.295	0.646–2.595	0.466			
HTN	2.413	1.353–4.304	0.003	2.159	1.233–3.783	0.007
Baseline eGFR	0.992	0.982–1.003	0.150			
Baseline Hb	0.986	0.875–1.112	0.822			
EBL (1st surgery)	1.000	0.999–1.001	0.799			
EBL (2nd surgery)	1.000	0.999–1.001	0.663			
Ischemic time (1st surgery)	1.005	0.974–1.037	0.763			
Ischemic time (2nd surgery)	0.987	0.957–1.019	0.431			
Pathologic tumor size (1st surgery)	1.015	1.006–1.024	0.001	1.012	1.003–1.021	0.010
Pathologic tumor size (2nd surgery)	1.004	0.996–1.011	0.326			
Time between operations	1.000	1.000–1.001	0.083			
Surgery sequence						
PN ➔ PN	Reference			Reference		
PN ➔ RN	2.249	1.088–4.786	0.006	1.837	1.028–3.635	0.007
RN ➔ PN	1.510	1.096–5.750	0.030	1.235	0.947–5.276	0.066
PN (unilateral case)	0.663	0.285–1.543	0.341	0.315	0.119–0.832	0.261
Tumor chronology						
Synchronous	Reference					
Metachronous	1.370	0.660–2.840	0.398			
Tumor multiplicity						
Unilateral	Reference					
Bilateral	1.244	0.698–2.215	0.459

**Table 6:** Multivariate Cox regression analyses of variables associated with de novo CKD during the entire follow-up period

	Univariate analysis			Multivariate analysis		
	HR	95% CI	*P* value	HR	95% CI	*P* value
Age (years)	1.031	1.009–1.053	0.005	1.005	0.971–1.040	0.780
BMI	1.029	0.946–1.120	0.500			
Sex, male	0.607	0.306–1.205	0.154			
DM	1.530	0.806–2.905	0.194			
HTN	1.956	1.147–3.335	0.014	1.905	1.172–3.265	0.010
Baseline eGFR	0.992	0.982–1.002	0.106			
Baseline Hb	1.002	0.902–1.114	0.969			
EBL (1st surgery)	1.000	0.999–1.000	0.784			
EBL (2nd surgery)	1.000	0.999–1.001	0.680			
Ischemic time (1st surgery)	1.001	0.972–1.032	0.921			
Ischemic time (2nd surgery)	0.985	0.956–1.014	0.301			
Pathologic tumor size (1st surgery)	1.016	1.007–1.025	0.001	1.013	1.000–1.027	0.051
Pathologic tumor size (2nd surgery)	1.004	0.997–1.011	0.278			
Time between operations	1.000	1.000–1.001	0.056			
Surgery sequence						
PN ➔ PN	Reference			Reference		
PN ➔ RN	2.919	1.386–6.146	0.001	1.888	1.088–4.055	< 0.001
RN ➔ PN	2.190	1.165–4.114	0.015	1.041	1.003–3.258	0.103
PN (unilateral case)	0.782	0.366–1.669	0.525	0.233	0.088–0.614	0.718
Tumor chronology						
Synchronous	Reference			Reference		
Metachronous	2.250	1.121–4.516	0.023	2.682	1.032–6.973	0.043
Tumor multiplicity						
Unilateral	Reference					
Bilateral	1.421	0.824–2.449	0.206			

## Discussion

Surgical management of RCC ultimately aims to balance numerous considerations with attempts to minimize operative morbidity and decline of renal function. To this end, when technically feasible, NSS has been advocated to maximize preservation of renal parenchyma and avoid the incidence and sequelae of renal function deterioration [19–22]. In the setting of bilateral renal masses, guidelines favor the performance of bilateral PN when it is technically feasible [10–12]. However, there is only a short reference to this simple guideline statement without specific details on how to do it in real clinical practice. For example, in case of the situation that both RN and PN are inevitably needed, it is a dilemma to decide which procedure has to be done first to achieve satisfactory renal function preservation. Nevertheless, using our prospectively collected database, we proved that doing RN first followed by PN sequence would be better in terms of preserving renal function than proceeding in reverse order. According to our multivariate analysis, hypertension and PN followed by RN sequence are commonly independent risk factors for de novo CKD within postoperative 1 year and the entire follow-up period. We also identified evidence in other literature supporting our findings. Krohn et al. have reported that 1 year after donation, the remained kidney manages to compensate up to 70% of renal function before surgery [23]. A plausible explanation is that in the remained kidney, vasodilation and increased renal plasma flow can occur immediately after surgery. These changes, combined with the process of glomerular hypertrophy, can increase glomerular filtration of the remnant kidney by approximately 40% without occurring a concomitant increase in glomerular capillary pressure [24–26]. This adaptive hyperfiltration also occurs in older kidney donors, although more modestly than in younger donors [27]. Taner et al. have reported that compensatory hypertrophy and GFR increase can occur in the remaining kidney of medically complex living donors at a comparable rate to those of standard donors [28]. These findings confirmed reassurance for delicately selected medically complex living donors. One study has concluded that RCC is not an independent risk factor for renal function decrease after nephrectomy. RCC patients with few morbidities could have the same deterioration of meanly 30% of kidney function compared with living donors. However, their lower baseline function can result in an increased risk for CKD [29]. These findings imply that patients with RN who have sufficient period to compensate for their renal function until PN can preserve favorable renal function without risk for CKD.

PN is generally related to a lower risk of developing clinically significant CKD than RN. Postoperative impairment in kidney function occurs most commonly in the first year after nephrectomy and appears stable over time. Age, Tumor stage, and preoperative kidney function are predictors of incident CKD after kidney cancer operation [30]. Bilateral PN needs more careful consideration compared to ipsilateral PN due to potential additional loss of renal function secondary to bilateral renal ischemia from hemorrhage, hypotension, and prolonged operative time [19]. These challenges have made some surgeons support staged bilateral PN as opposed to simultaneous bilateral PN in a single setting. PN, if performed well, is also a possible choice for larger renal tumors as it generates tolerable surgical morbidity, better renal function preservation, and equivalent cancer control with potential for better long-term survival than RN [31]. Bercz et al. showed acceptable oncological and functional outcomes from 65 patients. They reported significant postoperative renal function deterioration (44.8% eGFR decrease for synchronous (mostly RN → PN) and 30.4% decrease for metachronous tumor (all RN → PN) after the second operation, respectively), but hemodialysis was rarely required. Compared to their results, we showed more eGFR preservation if RN → PN (23.1% decrease) is performed respectively even though their cohort characteristics are slightly different [32].

This study has some limitations. First, even with two large tertiary centers’ cohort, the study population was still small due to the rarity of bilateral RCCs with its retrospective nature. In addition, there was no analysis of differences in renal functions that might occur due to the heterogeneity of surgical techniques and different time intervals of operation for bilateral metachronous RCC. Thirdly, our result from eight patients that PN followed by RN can negatively affect renal function preservation is necessary to be verified in further studies with larger numbers of patients. Finally, it was difficult to identify genetic differences between these patients. These issues can be elucidated if multicenter prospective randomized clinical trials are performed in the near future. Nevertheless, to the best of our knowledge, the current study is the first of its kind that assesses functional aspects of both kidney cancer surgery performed in large institutions over a relatively long period of time. This is also the first study to find that RN followed by PN is superior in terms of preserving renal functions in the opposite order by evaluating cohorts through propensity score matching analysis.

## Conclusion

This study evaluated renal functional outcomes of bilateral RCC patients after sequential kidney cancer surgery in a large contemporary cohort. The sequence of surgery should be prudently determined in bilateral renal tumors. The best ideal scenario is PN for both tumors. However, it is essential to make decisions about surgery sequence in the inevitable situation of performing a combination of radical and partial nephrectomy. PN followed by RN showed a negative impact on renal functional preservation. Thus, NSS should be considered for all amenable bilateral RCCs.

## Supplementary Information

**Additional file 1: Supplementary Table 1.** Baseline characteristics before propensity score matching.

## Data Availability

The datasets used and /or analyzed during the current study are available from the corresponding on reasonable request.

## Abbreviations

ADPKD: Autosomal dominant polycystic kidney disease
BHD: Birt-Hogg-Dubé syndrome
BMI: Body mass index
CKD: Chronic kidney disease
DM: Diabetes mellitus
EBL: Expected blood loss
eGFR: Estimated glomerular filtration rate
HTN: Hypertension
NSS: Nephron sparing surgery
PN: Partial nephrectomy
PSM: Propensity score matching
RCC: Renal cell carcinoma
RN: Radical nephrectomy
VHL: Von Hippel-Lindau disease
