# Supplementary material for: Evaluation of functional outcome of bilateral kidney tumors after sequential surgery
Source: BMC Cancer. 2021 May 24;21:592. doi: 10.1186/s12885-021-08324-3 (PMC8142506; doi:10.1186/s12885-021-08324-3)
Supplement: Supplementary file 1 — Additional file 1: Supplementary Table 1. Baseline characteristics before propensity score matching. [file 12885_2021_8324_MOESM1_ESM.docx]

Supplementary table 1. Baseline characteristics before propensity score matching

| Variables | Bilateral RCC  (N=178 lesions of 89 patients) | | Unilateral RCC  (N=2177 patients) | p value^§^ |
| --- | --- | --- | --- | --- |
|  | Synchronous  (N= 88) | Metachronous (N=90) |  |  |
| Age, mean (SD) | 54.0 ± 12.7 | 55.9 ± 12.9 | 55.2 ± 13.1 | 0.602 |
| Sex, male, N (%) | 74 (84.1%) | 74 (82.2%) | 1505 (69.1%) | < 0.001 |
| DM, yes, N (%) | 17 (19.3%) | 18 (20.0%) | 341 (15.7%) | 0.372 |
| HTN, yes, N (%) | 47 (53.4%) | 42 (46.7%) | 867 (39.8%) | 0.019 |
| BMI, mean (SD) | 25.0 ± 3.7 | 24.2 ± 3.1 | 24.6 ± 3.4 | 0.398 |
| ECOG performance status, N (%) |  |  |  | 0.259 |
| ≤ 1 | 86 (97.7%) | 86 (95.6%) | 2106 (96.7%) |  |
| ≥ 2 | 2 (2.3%) | 4 (4.4%) | 71 (3.3%) |  |
| Surgical type, N (%)  (Open / Laparoscopic / Robotic) | 37 (42.0%) /  13 (14.8%) /  38 (43.2%) | 44 (48.9%) /  9 (10.0%) /  37 (41.1%) | 801 (36.8%) /  249 (11.4%) /  1127 (51.8%) | 0.094 |
| EBL, ml, mean (SD) | 249.1 ± 107.5 | 259.0 ± 217.9 | 236.4 ± 413.8 | < 0.001 |
| Ischemic time, min, mean (SD)* | 21.5 ± 9.8 | 22.2 ± 10.7 | 20.7 ± 8.7 | 0.386 |
| Pathologic tumor size, mm, mean (SD) | 33.5 ± 34.8 | 32.1 ± 27.6 | 41.6 ± 30.5 | 0.001 |
| Baseline eGFR, ml/min/1.73 m^2^, mean (SD) | 71.6 ± 32.5 | 76.0 ± 25.4 | 83.9 ± 24.6 | < 0.001 |
| Baseline CKD, stage ≥ 3 | 28 (31.8%) | 16 (17.8%) | 254 (11.7%) | < 0.001 |

* Partial nephrectomy only.

^§^ Evaluates differences among all 3 groups
